# Lipoprotein(a): A Residual Cardiovascular Risk Factor in Statin-Treated Stroke Survivors: Insights From the SPARCL Trial

**DOI:** 10.1016/j.jacadv.2023.100557

**Published:** 2023-08-22

**Authors:** Kévin Chemello, Antonio Gallo, Alexis F. Guedon, Romuald Techer, Mikael Croyal, Michael J. Swietek, Olivier Meilhac, Pierre Amarenco, Gilles Lambert

**Affiliations:** a Inserm, UMR1188 Diabète athérothrombose Thérapies Réunion Océan Indien (DéTROI), Université de La Réunion, Sainte-Pierre, France; b Department of Endocrinology and Prevention of Cardiovascular Disease, Institute of Cardio Metabolism and Nutrition (ICAN), Sorbonne Université Pitié-Salpêtrière Hospital, SU-APHP, Paris, France; c APHP, Service de Médecine Interne, Département Hospitalo-Universitaire Inflammation Immunopathologie Biothérapie (DMUi3), Sorbonne Université, Paris, France; d CHU Nantes, CNRS, Inserm, BioCore, US16, SFR Bonamy, Nantes Université, Nantes, France; e CHU Nantes, CNRS, Inserm, Institut du Thorax, Nantes Université, Nantes, France; f CRNH-Ouest Mass Spectrometry Core Facility, Nantes, France; g Pfizer Inc, Groton, Connecticut, USA; h CHU de La Réunion, Saint-Denis, France; i INSERM 1148, Bichat Stroke Centre, Paris Université, Paris, France

**Keywords:** atorvastatin, CAD, lipoprotein(a), residual risk, stroke

## Abstract

**Background:**

In the SPARCL (Stroke Prevention by Aggressive Reduction in Cholesterol levels) trial, atorvastatin (80 mg/d) was compared to placebo in patients with recent stroke or transient ischemic attack (TIA) and no known coronary artery disease.

**Objectives:**

This study aimed to assess the contribution of lipoprotein(a) [Lp(a)] to subsequent cerebrovascular and cardiovascular events in stroke/TIA survivors.

**Methods:**

Lp(a) levels and apolipoprotein(a) [apo(a)] isoform size were determined by liquid-chromatography mass spectrometry in samples collected at baseline from 2,814 SPARCL participants (1,418 randomized to atorvastatin and 1,396 to placebo). Within each treatment arm, patients in the highest quartile (≥84.0 nmol/L) were compared with those in the lowest quartiles of Lp(a) concentrations. Patients in the lowest quartile (≤25.9 Kringle IV domains) of apo(a) isoform sizes were compared with those in the highest quartiles. Multivariable-adjusted HRs were calculated using Cox proportional regression models.

**Results:**

There was no significant association between Lp(a) concentrations or apo(a) isoform sizes and the risk of recurrent stroke, the primary outcome of SPARCL, or cerebrovascular events in patients randomized to atorvastatin or placebo. In contrast, in patients randomized to atorvastatin, elevated Lp(a) concentrations and short apo(a) isoforms were positively and independently associated with an increased risk of coronary events (HR: 1.607 [95% CI: 1.007-2.563] and HR: 2.052 [95% CI: 1.303-3.232]). No such association was found in patients randomized to placebo (HR: 1.025 [95% CI: 0.675-1.555] and HR: 1.097 [95% CI: 0.735-1.637]).

**Conclusions:**

Lp(a) contributes to the residual coronary artery disease risk of statin-treated stroke/TIA survivors, paving the way for use of therapies targeting Lp(a) in this population with stroke. (Lipitor In The Prevention Of Stroke, For Patients Who Have Had A Previous Stroke [SPARCL]; NCT00147602)

Lipoprotein(a) [Lp(a)] is a peculiar lipoprotein containing a unique glycoprotein, apolipoprotein(a) [apo(a)], covalently bound to the apolipoprotein B100 (apoB100) moiety of a low-density lipoprotein sized particle.^1^ Apo(a) is the product of the *LPA* gene and presents a highly repetitive structure consisting in 10 subtypes of the plasminogen-derived Kringle IV domain (KIV_1_-KIV_10_). The KIV_2_ domain is encoded by a pair of exons that can be repeated 1 to 40 times per allele.^2^ As a result, the size of apo(a) is highly polymorphic, its molecular weight ranging from 300 to 800 kDa. The size of apo(a) is inversely correlated with plasma Lp(a) concentrations, and explains 30% to 70% of Lp(a) variability.

Several observational and genetic studies have demonstrated that elevated Lp(a) levels are independently and significantly associated with coronary artery disease (CAD)^3^^,^^4^ and aortic valve stenosis.^5^^,^^6^ The association between Lp(a) and the risk of stroke is however less clear. No significant association was found between Lp(a) concentrations and the risk of stroke in 3 primary prevention cohorts.^7^, ^8^, ^9^ In contrast, high Lp(a) levels significantly associated with ischemic stroke in the ARIC (Atherosclerosis Risk In Communities) study^10^ as well as in a meta-analysis of 36 prospective studies.^11^ More recently, a seminal study conducted in a much larger population from Denmark also found a positive association between Lp(a) and ischemic stroke, both observationally and genetically.^12^ Importantly, the risk of CAD in this population was shown to increase above the 75th percentile of Lp(a) concentrations,^3^ whereas the risk of ischemic stroke only increased at much higher concentrations, ie, above the 95th percentile.^12^

Likewise, in secondary prevention, elevated Lp(a) levels appear to predict subsequent cardiovascular events in patients with established CAD.^13^^,^^14^ It remains to be seen whether Lp(a) also increases the cardiovascular risk specifically in patients with established cerebrovascular disease. The SPARCL (Stroke Prevention by Aggressive Reduction in Cholesterol levels) trial has demonstrated the superiority of 80 mg atorvastatin to placebo for the prevention of stroke in patients with recent stroke or transient ischemic attack (TIA).^15^^,^^16^ The aim of the current study was to investigate whether Lp(a) is predictive of recurrent cerebrovascular and incident coronary events in these patients.

## Methods

### Study population

Details of the SPARCL study protocol, efficacy and safety outcomes have been published previously.^15^^,^^16^ Briefly, 4,731 individuals aged ≥18 years with no known coronary heart disease and low-density lipoprotein-cholesterol (LDL-C) plasma concentrations between 100 and 190 mg/dL (2.6 and 4.9 mmol/L) were enrolled 1 to 6 months after a noncardioembolic stroke or TIA. Of a total of 4,731 SPARCL participants who gave written informed consent for the biomarker study, 2,814 (59.5%) had available plasma collected at baseline in sufficient quantities to determine apo(a) plasma concentrations and apo(a) size polymorphism by liquid-chromatography high-resolution mass spectrometry (Supplemental Methods). Presence of carotid stenosis at baseline was documented for 616 of the 2,814 patients (21.9%)^17^ (Supplemental Methods). Of these 2,814 patients, 1,418 (50.4%) were randomized to atorvastatin and 1,396 (49.6%) to placebo.

### Outcomes

The primary outcome was time from randomization to a first occurrence of a nonfatal or fatal stroke.^15^ Stroke was defined clinically by a sudden onset of focal neurologic symptoms related to impaired cerebral circulation lasting more than 24 hours, with or without neuroimaging evidence. Cerebral infarction and cerebral hemorrhage were defined by sudden onset of focal neurologic symptoms with: 1) evidence of cerebral ischemia on magnetic resonance imaging or computed tomography scan; or 2) neuroimaging evidence of bleeding in the appropriate area in the brain on magnetic resonance imaging or computed tomography scan, respectively. Secondary outcomes included time to cerebrovascular events (*defined as the occurrence of any ischemic stroke, hemorrhagic stroke, TIA, or carotid revascularization*), time to coronary events (*defined as the occurrence of any myocardial infarction, resuscitated cardiac arrest and other acute coronary events, coronary revascularization, unstable angina, and angina/ischemia requiring hospitalization*) and time to peripheral arterial events (*revascularization, peripheral artery disease*).^15^^,^^16^

### Statistical analysis

Statistical analyses were performed using SPSS version 23.0 (IBM) and R version 4.2.2 for Mac (Foundation for Statistical Computing). Normality of data distribution was determined using the Kolmogorov-Smirnov test. Categorical variables are expressed as counts and percentages. Symmetrically distributed quantitative variables are expressed as mean ± SD and not symmetrically distributed quantitative variables as median (interquartile range). Given that 80 mg atorvastatin starkly reduced the incidence of cerebrovascular, coronary, and peripheral vascular events in SPARCL,^15^ statistical analyses of the associations between Lp(a) and events were performed separately in the group randomized to atorvastatin and in the group randomized to placebo. In both groups, patients were stratified firstly according to their Lp(a) plasma concentrations at baseline by quartiles (Q1-Q3 vs Q4, cutoff value 84 nmol/L), and secondly according to the average size of their apo(a) isoforms by quartiles (Q1 vs Q2-Q4, cutoff value 25.9 KIV domains). Patients’ demographics and clinical characteristics were compared using a chi-square test for categorical variables, a Student’s *t*-test for parametric continuous variables, and a Mann-Whitney test for nonparametric continuous variables. A Spearman correlation coefficient was determined to verify the negative association between Lp(a) concentrations and apo(a) size.

Kaplan-Meier curves were used to generate cumulative incidence of primary and secondary outcomes and their components. The HRs (95% CIs) establishing the relationships between Lp(a) concentrations or apo(a) sizes and outcomes were determined using Cox proportional hazard models with no adjustment (Model 1), after adjustment for age and sex (Model 2), after adjustment for age, sex, type of entry event (stroke or TIA), and time since entry event (in days as a continuous variable), as in all previous SPARCL analyses^15^^,^^16^^,^^18^ (Model 3), and additionally for body mass index (BMI), non–high-density lipoprotein cholesterol (HDL-C) levels, smoking status, arterial hypertension, and diabetes at baseline (Model 4), that also are potential confounders. These parameters are those significantly varying in at least 1 treatment group when patients were stratified by quartile of Lp(a) concentrations or apo(a) size (Table 1), with non-HDL-C accounting for total cholesterol, LDL-C, apoB100, and triglycerides. Proportional-hazards assumptions were tested graphically using scaled Schoenfeld residuals.

**Table 1:** Baseline Characteristics by Treatment Arm Stratified by Quartile of Lp(a) Concentrations or apo(a) Size Distribution

	Lp(a) Concentrations						Apo(a) Size Distribution					
	Atorvastatin 80 mg (n = 1,418)			Placebo (n = 1,396)			Atorvastatin 80 mg (n = 1,418)			Placebo (n = 1,396)		
	<84 nmol/L (Q1-Q3) (n = 1,053)	≥84 nmol/L (Q4) (n = 365)	*P* Value	<84 nmol/L (Q1-Q3) (n = 1,058)	≥84 nmol/L (Q4) (n = 338)	*P* Value	>25.9 KIV (Q2-Q4) (n = 1,067)	≤25.9 KIV (Q1) (n = 351)	*P* Value	>25.9 KIV (Q2-Q4) (n = 1,044)	≤25.9 KIV (Q1) (n = 352)	*P* Value
Demographic												
Age (y)	63.6 ± 11.3	63.9 ± 10.8	NS	63.5 ± 11.3	62.2 ± 11.4	NS	63.6 ± 11.4	63.8 ± 10.6	NS	63.7 ± 11.3	61.5 ± 11.5	0.003
Male	651 (61.8)	195 (53.4)	0.005	625 (59.1)	183 (54.1)	NS	641 (60.1)	205 (58.4)	NS	592 (56.7)	216 (61.4)	NS
BMI (kg/m^2^)	27.6 ± 4.5	27.8 ± 5.3	NS	27.6 ± 4.7	27.5 ± 4.8	NS	27.5 ± 4.7	28.0 ± 4.8	0.034	27.4 ± 4.7	27.9 ± 4.6	NS
Risk factors												
Hypertension	625 (59.4)	250 (68.5)	0.002	675 (63.8)	188 (55.6)	0.007	651 (61.0)	224 (63.8)	NS	673 (64.5)	190 (54.0)	<0.001
Diabetes	177 (16.8)	57 (15.6)	NS	170 (16.1)	56 (16.6)	NS	179 (16.8)	55 (15.7)	NS	176 (16.9)	50 (14.2)	NS
Smoking	192 (18.2)	64 (17.5)	NS	210 (19.8)	60 (17.8)	NS	196 (18.4)	60 (17.1)	NS	195 (18.7)	75 (21.3)	NS
Lipoprotein(a)												
Lp(a) (nmol/L)	19.8 (9.4-41.6)	142 (107-201)	<0.001	21.1 (10.1-39.9)	137 (106-208)	<0.001	21.8 (10.5-53.1)	107 (52-172)	<0.001	22.4 (10.7-49.8)	98 (53-177)	<0.001
KIV domains (n)	34.9 ± 7.8	25.7 ± 6.0	<0.001	34.7 ± 8.0	24.9 ± 5.1	<0.001	35.9 ± 6.7	22.3 ± 3.3	<0.001	35.8 ± 6.8	22.3 ± 3.4	<0.001
Lipids (mg/dL)												
TC	210 ± 28	219 ± 31	<0.001	211 ± 29	216 ± 29	0.001	212 ± 29	213 ± 30	NS	212 ± 30	214 ± 29	NS
LDL-C	132 ± 23	139 ± 24	<0.001	133 ± 25	138 ± 24	0.002	134 ± 23	134 ± 24	NS	134 ± 24	136 ± 24	NS
HDL-C	50 ± 13	52 ± 15	0.008	50 ± 14	51 ± 14	NS	51 ± 14	50 ± 14	NS	50 ± 14	49 ± 13	NS
TG	139 ± 65	139 ± 65	NS	142 ± 68	137 ± 70	NS	137 ± 62	148 ± 71	0.005	139 ± 66	146 ± 77	NS
Apolipoproteins (mg/dL)												
ApoA-I	149 ± 27	152 ± 30	NS	148 ± 27	149 ± 29	NS	150 ± 28	149 ± 29	NS	149 ± 28	147 ± 28	NS
ApoB100	132 ± 21	137 ± 22	0.001	134 ± 23	137 ± 21	0.029	133 ± 21	135 ± 22	NS	134 ± 22	137 ± 22	0.007

Values are mean ± SD, n (%), or median (IQR).

apo(a) = apolipoprotein(a); ApoB100 = apolipoprotein B100; BMI = body mass index; HDL = high-density lipoprotein; KIV = kringle IV; LDL = low-density lipoprotein; Lp(a) = lipoprotein(a); TC = total cholesterol; TG = triglycerides.

Composite cardiovascular end points were categorized according to the vascular territories (see above). Since similar results were obtained with models 1, 2, 3, and 4, only HR (95% CI) and their *P* values determined using stratified log-rank tests established with model 4 are displayed. Sensitivity analyses were performed using inverse probability treatment weighting (IPTW) to adjust for differences in baseline covariates between Lp(a) concentrations and apo(a) size groups^19^^,^^20^ (Supplemental Methods). Potential confounders included in the logistic regression model used to estimate propensity scores included the following covariates at baseline: age, sex, BMI, smoking status, arterial hypertension, diabetes, non-HDL-C, presence of carotid stenosis, type of entry event (Stroke or TIA), and time since entry event (in days). IPTW-adjusted HR (95% CI) for outcomes were estimated using Cox proportional-hazards models. Balances between groups were assessed by the standardized differences of all baseline covariates, using a threshold of 0.1 to indicate an imbalance.^21^ We also performed Fine-Gray models to account for death as a competing risk and obtain subdistribution HR for primary and secondary outcomes.^22^ Two-tailed *P* values <0.05 were considered significant with no adjustment for multiple testing in these exploratory analyses.

## Results

Lp(a) concentrations and apo(a) isoforms sizes were measured in plasma samples collected at baseline from 2,814 SPARCL participants. Their demographic, clinical and biological characteristics (Supplemental Table 1) were not significantly different from those reported for the 4,731 participants of the entire SPARCL cohort.^15^ There was no significant difference between the 1,418 patients later randomized to atorvastatin and the 1,396 patients later randomized to placebo for any of these parameters, including Lp(a) concentration and apo(a) size (Supplemental Table 1). As anticipated, the distribution of Lp(a) concentrations among the 2,814 SPARCL participants was skewed (Supplemental Figure 1) around a median of 32.1 (IQR: 12.9-84.0) nmol/L, with a negative correlation (r = −0.639; *P* < 0.001) between circulating Lp(a) levels and the size of apo(a) isoforms.

The atorvastatin and placebo groups were stratified by quartiles of Lp(a) plasma concentration at baseline (Table 1). In the atorvastatin group, patients from the highest Lp(a) quartile (≥84 nmol/L) were more likely to be women, hypertensive and presented with higher HDL-C levels. In the placebo group, the prevalence of hypertension was reduced in patients from the highest Lp(a) quartile. In both groups, the circulating levels of total cholesterol, LDL-C and apoB100 were increased in patients in the highest quartile of Lp(a) concentrations. We also stratified both treatment groups by quartiles of apo(a) size distribution (Table 1). In the atorvastatin group, patients BMIs and plasma triglycerides were higher in the bottom quartile of apo(a) isoform sizes (≤25.9 KIV domains). In the placebo group, patients were significantly younger, less hypertensive, and had higher levels of apoB100 in the bottom quartile of apo(a) isoform sizes.

### Cerebrovascular events

The association between Lp(a) concentration or apo(a) size and a first nonfatal or fatal subsequent stroke, the primary end point of SPARCL, was evaluated.^15^^,^^16^ Over a median follow-up period of 53 (IQR: 24-60) months, 169 and 176 strokes occurred in the atorvastatin and placebo groups, representing 11.9% and 12.6% of patients, respectively. Neither elevated Lp(a) concentrations nor short apo(a) isoforms were associated with the risk of subsequent stroke in both the atorvastatin (HR: 0.776 [95% CI: 0.540-1.116]; *P* = 0.172 and HR: 1.053 [95% CI: 0.693-1.395]; *P* = 0.925) and placebo (HR: 1.005 [95% CI: 0.708-1.426]; *P* = 0.979 and HR: 1.011 [95% CI: 0.741-1.495]; *P* = 0.604, respectively) groups.

**Figure 1:**
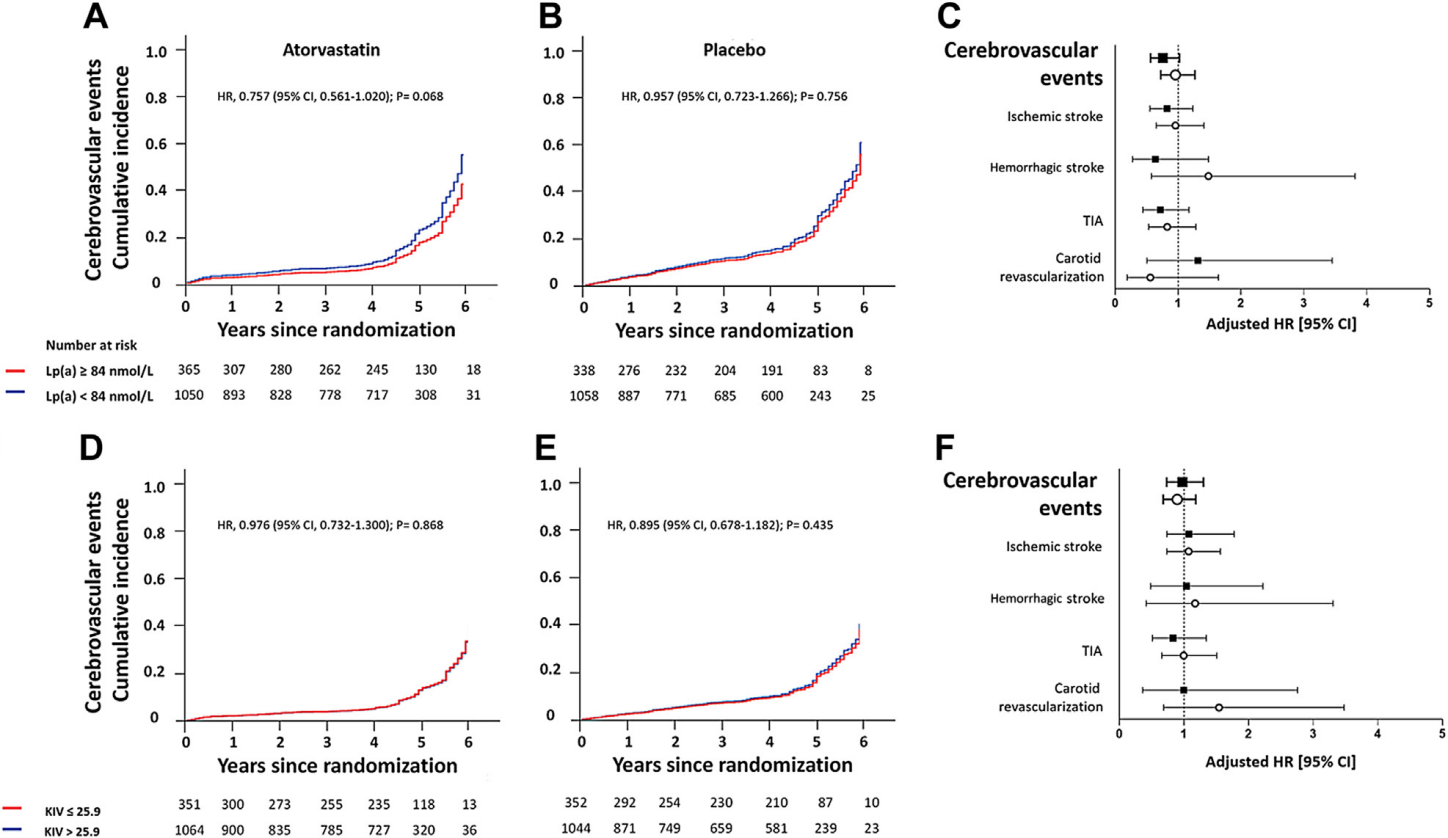
Cumulative Incidence and HRs of Cerebrovascular Events by Levels of Lp(a) Plasma Concentrations at Baseline and by Levels of Apo(a) Size Distribution During 6 Years Following Randomization Kaplan-Meier estimates for cerebrovascular events in patients randomized to atorvastatin 80 mg **(A)** or placebo **(B)** with lipoprotein(a) plasma concentrations below (quartiles 1-3) or above (quartile 4) 84 nmol/L at baseline. **(C)** HRs for composite cerebrovascular events and each major type of cerebrovascular event by levels of lipoprotein(a) plasma concentrations at baseline in patients randomized to atorvastatin 80 mg **(black squares)** or placebo **(open circles)**. Kaplan-Meier estimates for cerebrovascular events in patients randomized to atorvastatin 80 mg **(D)** or placebo **(E)** with apolipoprotein(a) size below (quartile 1) or above (quartiles 2-4) 25.9 Kringle IV domains. **(F)** HRs for composite cerebrovascular events and each major type of cerebrovascular event by apolipoprotein(a) size in patients randomized to atorvastatin 80 mg **(black squares)** or placebo **(open circles)**. Kaplan-Meier estimates and HRs were adjusted for age, sex, type of entry event, time to entry event, body mass index, non–high-density lipoprotein cholesterol levels, smoking, hypertension, and diabetes at baseline. KIV = Kringle IV; Lp(a) = lipoprotein(a); TIA = transient ischemic attack.

We also evaluated the associations between Lp(a) concentrations or apo(a) sizes and all subsequent cerebrovascular events. Over the follow-up period, a total of 264 and 294 cerebrovascular events occurred in the atorvastatin and placebo groups, representing 18.6% and 21.1% of patients, respectively (Supplemental Tables 2 and 3). Likewise, neither elevated Lp(a) concentrations nor short apo(a) isoforms significantly associated with the risk of subsequent cerebrovascular events in both the atorvastatin (HR: 0.757 [95% CI: 0.561-1.020]; *P* = 0.068 and HR: 0.976 [95% CI: 0.732-1.300]; *P* = 0.868), and placebo (HR: 0.957 [95% CI: 0.723-1.266]; *P* = 0.756 and HR: 0.895 [95% CI: 0.678-1.182]; *P* = 0.435) groups, respectively. Kaplan-Meier curves on the basis of quartiles of Lp(a) plasma concentration and apo(a) size illustrate the lack of association between both parameters and any subtype of cerebrovascular events in both treatment arms of SPARCL (Figure 1). Similar analyses were performed in the subgroup of patients with carotid stenosis at baseline (Supplemental Tables 4 and 5). As in the entire study population, neither elevated Lp(a) concentrations nor short apo(a) isoforms significantly associated with the risk of subsequent cerebrovascular events (including ischemic stroke) in both the atorvastatin (HR: 1.314 [95% CI: 0.737-2.343]; *P* = 0.355, and HR: 1.278 [95% CI: 0.719-2.271]; *P* = 0.403) and placebo (HR: 0.692 [0.385-1.242]; *P* = 0.217 and HR: 0.946 [95% CI: 0.526-1.701]; *P* = 0.853) groups, respectively.

### Coronary events

**Figure 2:**
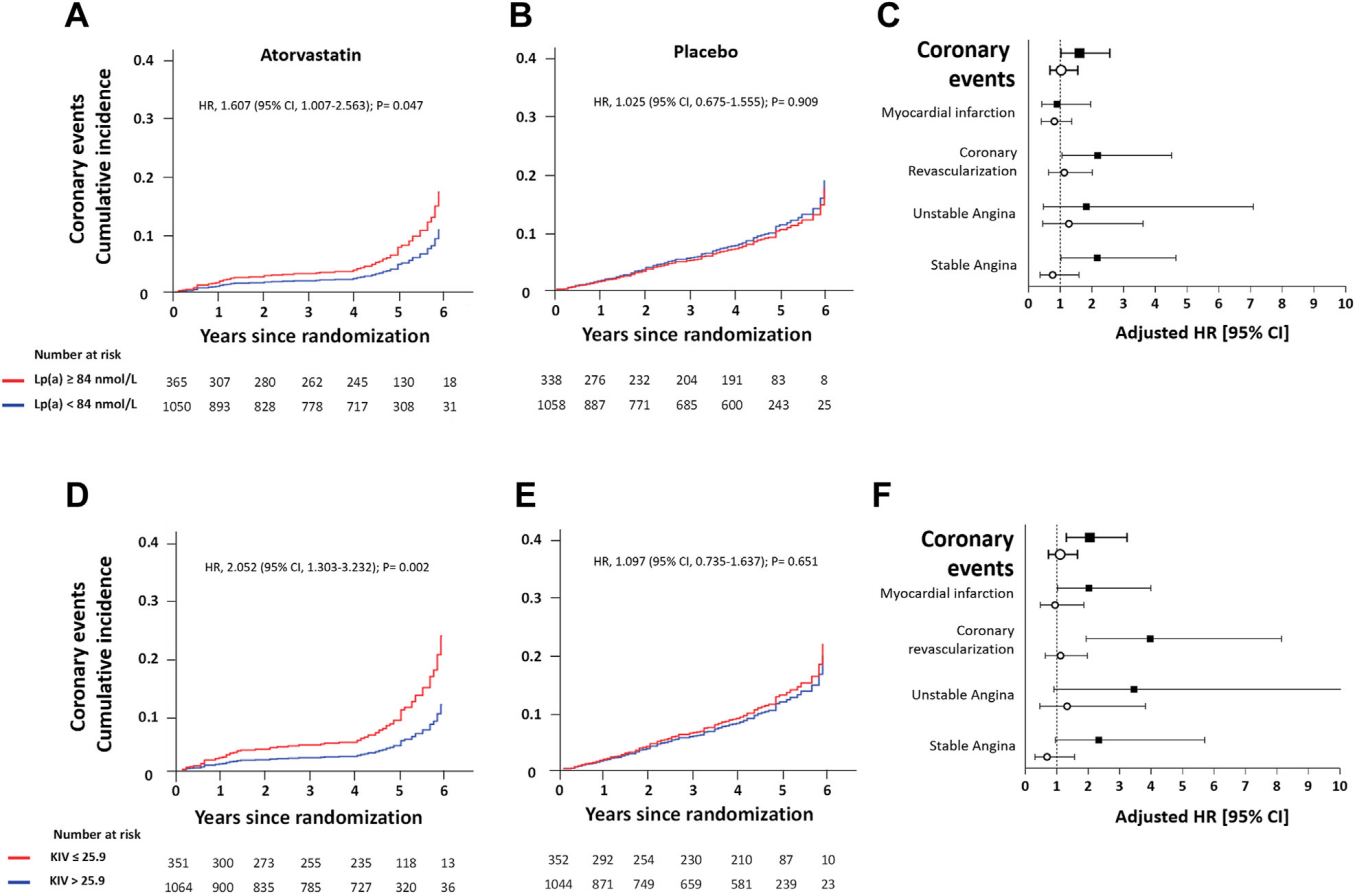
Cumulative Incidence and HRs of Coronary Events by Levels of Lp(a) Plasma Concentrations at Baseline and by Levels of Apo(a) Size Distribution During 6 Years Following Randomization Kaplan-Meier estimates for coronary events in patients randomized to atorvastatin 80 mg **(A)** or placebo **(B)** with lipoprotein(a) plasma concentrations below (quartiles 1-3) or above (quartile 4) 84 nmol/L at baseline. **(C)** HRs for composite coronary events and each major type of coronary event by levels of lipoprotein(a) plasma concentrations at baseline in patients randomized to atorvastatin 80 mg **(black squares)** or placebo **(open circles)**. Kaplan-Meier estimates for coronary events in patients randomized atorvastatin 80 mg **(D)** or placebo **(E)** with apolipoprotein(a) size below (quartile 1) or above (quartiles 2-4) 25.9 Kringle IV domains. **(F)** HRs for composite coronary events and each major type of coronary event by apolipoprotein(a) size in patients randomized to atorvastatin 80 mg **(black squares)** or placebo **(open circles)**. Kaplan-Meier estimates and HRs were adjusted for age, sex, type of entry event, time to entry event, body mass index, non–high-density lipoprotein cholesterol levels, smoking, hypertension, and diabetes at baseline. KIV = Kringle IV; Lp(a) = lipoprotein(a).

The associations between Lp(a) concentrations or apo(a) sizes and all incident coronary events was evaluated. Over the follow-up period, 78 and 124 coronary artery events occurred in the atorvastatin and placebo groups, representing 5.5% and 8.9% of patients, respectively (Supplemental Tables 2 and 3). In the atorvastatin group, elevated Lp(a) concentrations and short apo(a) isoform sizes were positively and significantly associated with an increased risk of coronary events (HR: 1.607 [95% CI: 1.007-2.563]; *P* = 0.047 and HR: 2.052 [95% CI: 1.303-3.232]; *P* = 0.002), respectively. Throughout, propensity scores analyses yielded similar IPTW-adjusted HR and subdistribution HR (accounting for deaths) for all outcomes (Supplemental Tables 6 and 7). Kaplan-Meier curves on the basis of quartiles of Lp(a) plasma concentration and apo(a) size illustrate the associations between both parameters and incident coronary events in the atorvastatin group (Figure 2). These associations were primarily driven by a significantly higher incidence of coronary revascularization procedures, angina/ischemia requiring hospitalization, and/or myocardial infarction (Supplemental Tables 2 and 3). In the placebo group, neither elevated Lp(a) concentrations nor short apo(a) isoforms associated with the risk of coronary events [HR: 1.025 [95% CI: 0.675-1.555]; *P* = 0.909 and HR: 1.097 [95% CI: 0.735-1.637]; *P* = 0.651), respectively (Figure 2).

### Peripheral vascular events

Over the follow-up period, 10 and 19 peripheral vascular events occurred in the atorvastatin and placebo groups, representing 0.7% and 1.4% of patients, respectively (Supplemental Tables 2 and 3). There was no significant association between Lp(a) or apo(a) size and incident peripheral events in either group (Supplemental Figure 2).

## Discussion

The SPARCL trial was the first to demonstrate that aggressive LDL-C lowering with 80 mg atorvastatin reduces the incidence of strokes, as well as major coronary events, and other cardiovascular events in patients with a recent history of symptomatic cerebrovascular disease.^15^^,^^16^ To evaluate if Lp(a) contributes to the residual risk of these patients, we measured Lp(a) concentration and the size of apo(a) isoforms in their plasma. We did not find any significant association between Lp(a) concentrations or apo(a) isoforms and the risk of recurrent stroke and cerebrovascular events in either treatment arm of the trial. In contrast, elevated Lp(a) concentrations and short apo(a) isoforms were associated with an increased risk of coronary events in patients treated with atorvastatin. No such association was found in patients randomized to placebo. A visual summary of the data is presented in the Central Illustration.

**Central Illustration:**
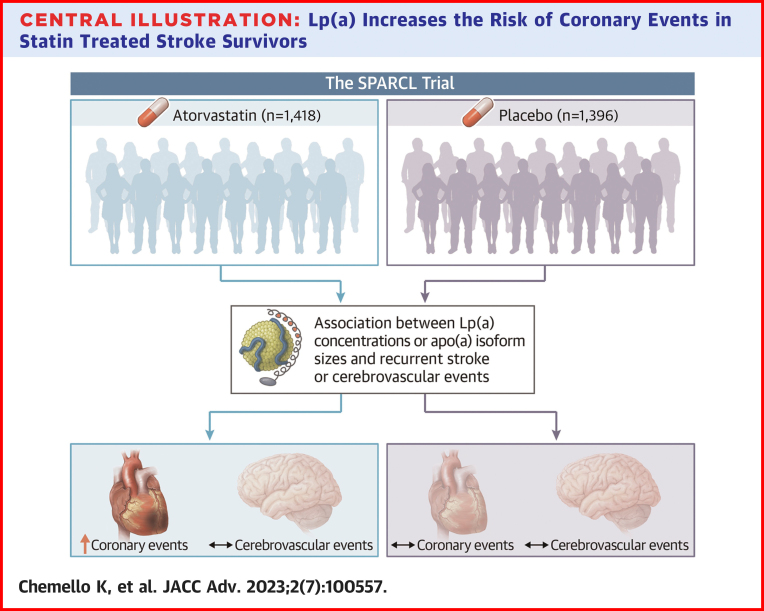
Lp(a) Increases the Risk of Coronary Events in Statin Treated Stroke Survivors Elevated lipoprotein(a) concentrations and short apolipoprotein(a) isoforms are predictors of subsequent coronary events but not of recurrent cerebrovascular events in patients in secondary prevention of stroke or transient ischemic attack on a background of statin therapy. apo(a) = apolipoprotein(a); Lp(a) = lipoprotein(a).

In the current study, no association between Lp(a) concentrations or the size of apo(a) isoforms and the risk of recurrent stroke or cerebrovascular events was found. This neutral result is not surprising given that in primary prevention only very high concentrations of Lp(a) associate with the risk of stroke, and that these associations are significant only when very large populations are studied.^4^^,^^11^^,^^12^ In line with our results, Lp(a) concentrations above 150 nmol/L increased the risk of ischemic stroke in primary prevention (HR: 1.16 [95% CI: 1.07-1.25]) but not in secondary prevention of cardiovascular diseases (HR: 0.93 [95% CI: 0.77-1.12]) in the UK biobank.^14^ Discrepant with our results though, Lp(a) above 50 mg/dL (≈125 nmol/L) was recently found associated with a 20% increased risk of recurrent stroke over follow-up period of 1 year among 9,899 stroke/TIA survivors from China.^23^ However, Lp(a)-increasing genetic variants that predict subsequent CAD failed to predict the risk of ischemic stroke in secondary prevention of cardiovascular disease in another study.^24^ Neither did elevated Lp(a) concentrations predict the risk of stroke in a small cohort of 250 stroke survivors.^25^ Noteworthy, Lp(a) was shown to only increase the risk of large artery atherosclerotic stroke but not that of lacunar or cardioembolic strokes in the ARIC study.^26^^,^^27^ A mendelian randomization study also showed that Lp(a) associates with an increased risk of large artery stroke but with a decreased risk of small vessel stroke.^28^ Furthermore, elevated Lp(a) was found associated with large artery atherosclerosis stroke etiology in acute ischemic stroke survivors.^29^ To determine whether Lp(a) might predict the risk of subsequent cerebrovascular events specifically in patients with established atherosclerosis, we specifically analyzed the subgroup of SPARCL participants with carotid stenosis at baseline.^17^ Albeit nonstatistically significant, the association between Lp(a) and cerebrovascular events that seemed negative in the entire study population (HR: 0.757 [95% CI: 0.561-1.020]) appeared positive in this subgroup (HR: 1.314 [95% CI: 0.737-2.343]), suggesting a possible contribution of Lp(a) to the residual cerebrovascular risk for patients with atherosclerosis. This clearly needs to be properly tested in a larger cohort of stroke survivors with documented evidence of large vessels atherosclerosis.^30^^,^^31^ In aggregate, the size of our cohort, the complex etiology or stroke, and the relatively limited effects of Lp(a) on cerebrovascular disease likely concur to the lack of association between Lp(a) and the risk of recurrent cerebrovascular events observed in the SPARCL study.

We found that Lp(a) predicts the risk of subsequent coronary event in the atorvastatin arm of SPARCL. This result extends the observations of post hoc analyses of proprotein convertase subtilisin kexin type 9 inhibitor (PCSK9i) trials, in which patients with established cardiovascular disease and treated with maximally tolerated doses of statins were randomized either to PCSK9i or placebo.^32^, ^33^, ^34^ In both trials, PCSK9i treatments reduced Lp(a) and the associated risk of subsequent coronary events.^32^, ^33^, ^34^ In contrast, we did not find any association between Lp(a) and coronary events in the placebo arm of SPARCL, suggesting that elevated LDL-C probably masks out the CAD risk specifically stemming from elevated Lp(a). This is corroborated by a post hoc analysis of the PCSK9i trials showing that patients with higher LDL-C levels derived similar clinical benefits from the treatment irrespective of their Lp(a) levels, whereas patients with LDL-C below target (70 mg/dL) derived incremental clinical benefits when their baseline Lp(a) was high.^35^

No association between Lp(a) and subsequent peripheral events in SPARCL was seen, a result at odds with those of the post hoc analysis of the PCSK9i trials.^36^ A likely explanation is a lack of statistical power, given that the number of peripheral events in SPARCL was extremely low.^16^ The results of our study are also at odds with a previous report showing that elevated concentrations of oxidized phospholipids associated with apolipoprotein B100 containing lipoproteins (OxPL-apoB) predict the recurrence of stroke and the incidence of coronary events in SPARCL.^37^ This biomarker is supposed to reflect Lp(a) concentrations, since Lp(a) is a major carrier of oxidized phospholipids,^37^, ^38^, ^39^ but this is challenged by the following observations: 1) OxPL-apoB levels measured in SPARCL participants were higher among diabetics,^37^ whereas Lp(a) is consistently reduced in this pathology;^40^^,^^41^ and 2) OxPL-apoB significantly decreased during follow-up,^37^ whereas Lp(a) levels are known to increase in response to high-intensity statins treatments.^42^^,^^43^

### Study limitations

Our study is a post hoc analysis that was conducted in 60% of SPARCL participants. The baseline characteristics of this large subgroup were similar to those of the entire cohort. The 2021 American Heart Association/American Stroke Association guidelines recommend to reduce LDL-C below 70 mg/dL, a level not reached by a significant proportion of SPARCL participants from the atorvastatin group.^15^ Addition of ezetimibe and eventually of a PCSK9i to high-intensity statin treatment is more representative of current clinical practice.^44^ The association between Lp(a) and coronary events was of borderline significance and primarily driven by the number of revascularization procedures as well as angina/ischemia requiring hospitalization or myocardial infarction. Overall the number of subsequent coronary events in SPARCL was small. Lastly, interleukin-6 a strong inducer of LPA gene expression^45^ and its downstream inflammatory marker, the C-reactive protein, that are known to modify the association between Lp(a) and events^23^^,^^46^ have not been measured in SPARCL.

## Conclusions

This study indicates that elevated Lp(a) levels contribute to the residual CAD risk of statin-treated patients with a history of stroke or TIA. These findings highlight the importance of systematically measuring Lp(a)^47^ and the protective role of high-intensity statin treatment in secondary prevention of stroke or TIA. In clinical development, antisense oligonucleotides^48^ and small interfering RNAs^49^^,^^50^ targeting apo(a) production, have both yielded >90% reductions in circulating Lp(a) concentrations. In addition to high doses statins, these novel agents, may help to reduce the CAD risk in patients with cerebrovascular disease and elevated Lp(a).

## Funding support and author disclosures

This work was supported by the 10.13039/501100001665*Agence Nationale de la Recherche* (Paris, France) Project Grant KRINGLE2 ANR-20-CE14-0009 and by *La Fondation De France* (Paris, France) Project Grant FDF-00096274. These agencies had no role in the design and conduct of the study, in the collection, analysis, and interpretation of the data, and in the preparation, review, or approval of the manuscript. Dr Chemello is supported by a scholarship from the *European Regional Development Fund & La Région Réunion* (Saint-Denis de La Réunion, France). Dr Gallo is supported by a postdoctoral fellowship SPF 202004011793 from *La Fondation Pour La Recherche Médicale* (Paris, France; and reports consultancy fees and honoraria from Akcea Therapeutics, Amgen, Mylan, Novartis, Sanofi, Unilever, and MSD. Dr Swietek is an employee of Pfizer. Dr Amarenco has received research grant support from Pfizer, Sanofi, BMS, Merck, AstraZeneca, Boston Scientific, Althera Pharmaceutical, and the French government; consulting fees from Pfizer, BMS, AstraZeneca, Bayer, Jansen, Kowa, Amgen, and Portola; and lecture fees from Amgen, Pfizer and Sanofi. Dr Lambert has received research grant support and consulting fees for serving on the scientific advisory board from Nyrada. All other authors have reported that they have no relationships relevant to the contents of this paper to disclose.

PERSPECTIVES **COMPETENCY IN MEDICAL KNOWLEDGE:** Statins are indicated for patients in secondary prevention of symptomatic cerebrovascular disease to reduce their risk of recurrent stroke or cardiovascular events. Our study demonstrates that even when high-intensity statin treatment is implemented, elevated levels of Lp(a) independently and significantly contribute to the residual CAD risk of these patients.

**TRANSLATIONAL OUTLOOK:** Therapies that lower Lp(a) (*currently limited to PCSK9i but in the future to antisense and siRNAs targeting Lp(a) production*) should be added on top of statins for patients in secondary prevention of stroke or TIA with elevated Lp(a).
