# Supplementary material for: Lipoprotein(a): A Residual Cardiovascular Risk Factor in Statin-Treated Stroke Survivors: Insights From the SPARCL Trial
Source: JACC Adv. 2023 Aug 22;2(7):100557. doi: 10.1016/j.jacadv.2023.100557 (PMC11198425; doi:10.1016/j.jacadv.2023.100557)
Supplement: Supplementary Data [file mmc1.docx]

**SUPPLEMENTAL APPENDIX**

**Supplemental Methods**

**Apolipoproteins measurement** - Plasma samples were prepared using a fully validated method ^1,2^ involving trypsin proteolysis and subsequent analysis of proteotypic peptides on a Dionex Ultimate 3000 UHPLC System interfaced to Q Exactive Plus Orbitrap mass spectrometer (Thermo Fisher Scientific, Sano Jose CA, USA) by targeted parallel reaction monitoring assay. Apo(a) proteotypic peptides were quantitated in molar units ^2^. As the target peptide LFLEPTQADIALLK is unique in apo(a), one mole of this peptide is assumed equal to one mole of apo(a) and one mole of apo(a) to one mole of Lp(a). As the target peptide GTYSTTVTGR is present in KIV_1_, KIV_2_, KIV_3_, and KIV_5_ but not in KIV_4_, and KIV_6_ to KIV_10_ domains, the total number of KIV domains on circulating apo(a) is equal to ([GTYSTTVTGR]_nM_ ÷ [LFLEPTQADIALLK]_nM_) + 6, and corresponds to the average size of apo(a) isoforms present in plasma ^3^.

**Subgroup with carotid stenosis** - Presence of carotid stenosis was documented for 616 of the 2,814 patients (21.9%). As previously reported ^4^, these patients were on average older and the proportion of males, smokers and diabetics was higher in this subgroup. Their plasma lipids, lipoproteins and apolipoproteins concentrations were similar to those of the entire study population (not shown). The proportion of individuals with carotid stenosis at entry was higher among patients in the highest quartile of Lp(a) concentrations (23.3% vs. 21.4%), and among patients in the lowest quartile of apo(a) size distribution (22.9% vs. 21.6%). In this subgroup of 616 patients, 59 and 81 cerebrovascular events, 35 and 52 ischemic strokes, as well as 25 and 35 coronary events occurred during follow-up in the atorvastatin and placebo groups, representing 19.8% and 25.4%, 11.7% and 16.3%, and 8.4% and 10.9% of patients, respectively.

**Inverse probability treatment weighting (IPTW)** - We used IPTW to adjust for differences in baseline covariates between Lp(a) and apo(a) quartiles groups. Although the word treatment is used in the propensity score literature to refer to exposure, the technique is applicable to any type of exposure, such as high Lp(a) levels or small apo(a) isoforms. This allowed comparison of event-free survival between groups after minimizing imbalances in prognostic variables. Potential confounders included in the logistic regression model used to estimate propensity scores included the following covariates at baseline: age, sex, body mass index, smoking status, hypertension, diabetes, non-HDL cholesterol levels, qualifying entry event (Stroke or TIA), presence of carotid stenosis at baseline, and time since entry event (in days). For Lp(a) concentrations analysis, patients were weighted by the stabilized inverse probability of being in the **Q4** group. For apo(a) size analysis, patients were weighted by the stabilized inverse probability of being in the **Q1** group. We derived 95% confidence intervals using a robust variance estimator. Balances between groups were assessed by the standardized differences of all baseline covariates, using a threshold of 0.1 to indicate an imbalance. All standardized differences in all models were below 0.1 (not shown).

**Supplemental Table 1 – Baseline Characteristics of the study population**

**

*Values displayed as means ± SE or median [interquartile range] for continuous variables and as percentages for categorical variables. TC denotes total cholesterol, LDL-C low-density lipoprotein cholesterol, HDL-C high-density lipoprotein cholesterol, and TG Triglycerides. The BMI denotes body-mass index and is the weight in kilograms divided by the square of the height in meters.*

**Supplemental Table 2 – Distribution and Hazard Ratios of coronary, cerebrovascular and peripheral events by treatment arm and by quartile of Lp(a) plasma concentrations at baseline.**

**

*Events are displayed as n (percentages). Hazard Ratios were adjusted for age, sex, type of entry event, time to entry event, BMI, non-HDL cholesterol levels, smoking, hypertension, and diabetes at baseline. HR denotes hazard ratios, CI confidence intervals, and KIV Kringle IV domains.*

|  | **Atorvastatin 80mg** | | | | | | | **Placebo** | | | | | | | | | | |
| --- | --- | --- | --- | --- | --- | --- | --- | --- | --- | --- | --- | --- | --- | --- | --- | --- | --- | --- |
|  | **> 25.9 KIV**  **(Q2-Q4)** | **≤ 25.9 KIV**  **(Q1)** | ***P*** | **HR** | **95%CI** | ***P*** | | | **> 25.9 KIV**  **(Q2-Q4)** | **≤ 25.9 KIV**  **(Q1)** | | ***P*** | | **HR** | **95%CI** | | ***P*** | |
|  | (n = 1067) | (n = 351) |  |  |  |  |  |  | (n = 1044) | (n = 352) | |  |  |  |  |  |  |  |
| **Coronary events** | 46 (4.3) | 32 (9.1) | **0.001** | 2.052 | 1.303-3.232 | | **0.002** | | 96 (9.2) | | 34 (9.7) | | 0.796 | 1.097 | | 0.735-1.637 | | 0.651 |
| Myocardial Infarction | 21 (2.0) | 14 (4.0) | **0.035** | 2.020 | 1.023-3.989 | | **0.043** | | 43 (4.1) | | 11 (3.1) | | 0.403 | 0.941 | | 0.476-1.863 | | 0.862 |
| Coronary revascularization | 13 (1.2) | 18 (5.2) | **<0.001** | 3.970 | 1.934-8.146 | | **<0.001** | | 47 (4.5) | | 18 (5.1) | | 0.583 | 1.117 | | 0.633-1.973 | | 0.703 |
| Unstable Angina | 4 (0.4) | 5 (1.4) | **0.032** | 3.454 | 0.911-13.098 | | 0.068 | | 14 (1.3) | | 5 (1.4) | | 0.911 | 1.326 | | 0.461-3.820 | | 0.601 |
| Angina | 19 (1.8) | 9 (2.6) | 0.361 | 2.334 | 0.955-5.702 | | 0.063 | | 34 (3.3) | | 12 (3.4) | | 0.890 | 0.690 | | 0.304-1.564 | | 0.374 |
| **Cerebrovascular events** | 199 (18.7) | 65 (18.5) | 0.880 | 0.976 | 0.732-1.300 | | 0.868 | | 228 (21.8) | | 66 (18.8) | | 0.219 | 0.895 | | 0.678-1.182 | | 0.435 |
| Ischemic Stroke | 101 (9.4) | 35 (9.9) | 0.502 | 1.076 | 0.735-1.775 | | 0.706 | | 120 (11.5) | | 36 (10.2) | | 0.549 | 1.071 | | 0.733-1.563 | | 0.724 |
| Hemorragic Stroke | 25 (2.3) | 8 (2.3) | 0.974 | 1.038 | 0.485-2.220 | | 0.923 | | 15 (1.4) | | 5 (1.4) | | 0.881 | 1.171 | | 0.415-3.306 | | 0.766 |
| Transcient Ischemic Attack | 81 (7.6) | 24 (6.8) | 0.637 | 0.829 | 0.511-1.344 | | 0.446 | | 96 (9.2) | | 32 (9.1) | | 0.953 | 0.995 | | 0.656-1.509 | | 0.981 |
| Carotid revascularisation | 15 (1.4) | 6 (1.7) | 0.684 | 0.996 | 0.360-2.758 | | 0.994 | | 19 (1.8) | | 9 (2.6) | | 0.394 | 1.542 | | 0.684-3.478 | | 0.297 |
| **Peripheral events** | 5 (0.5) | 5 (1.4) | 0.064 | 2.797 | 0.783-9.995 | | 0.113 | | 17 (1.6) | | 2 (0.6) | | 0.138 | 0.415 | | 0.094-1.830 | | 0.245 |

**Supplemental Table 3 – Distribution and Hazard Ratios of coronary, cerebrovascular and peripheral events by treatment arm and by quartile of apo(a) size.**

*Events are displayed as n (percentages). Hazard Ratios were adjusted for age, sex, type of entry event, time to entry event, BMI, non-HDL cholesterol levels, smoking, hypertension, and diabetes at baseline. HR denotes hazard ratios, CI confidence intervals, and KIV Kringle IV domains.*

**Supplemental Table 4 - Baseline characteristics by treatment arm stratified by quartiles of Lp(a) concentrations or apo(a) size distribution in the subgroup of 616 patients with carotid stenosis at baseline.**

|  | | **Lp(a) concentrations** | | | | | | | | **Apo(a) size distribution** | | | | | | | |
| --- | --- | --- | --- | --- | --- | --- | --- | --- | --- | --- | --- | --- | --- | --- | --- | --- | --- |
|  | | **Atorvastatin 80 mg**  **(n=297)** | | | | **Placebo**  **(n=319)** | | | | **Atorvastatin 80 mg**  **(n=297)** | | | **Placebo**  **(n=319)** | | | |  |
|  |  | **< 84 nmol/L**  **(Q1-Q3)**  **(n = 219)** | **≥ 84 nmol/L**  **(Q4)**  **(n = 78)** | ***P*** | **< 84 nmol/L**  **(Q1-Q3)**  **(n = 233)** | | **≥ 84 nmol/L**  **(Q4)**  **(n = 86)** | ***P*** | **> 25.9 KIV**  **(Q2-Q4)**  (n = 217) | | **≤ 25.9 KIV**  **(Q1)**  (n = 80) | ***P*** | | **> 25.9 KIV**  **(Q2-Q4)**  (n = 238) | **≤ 25.9 KIV**  **(Q1)**  (n = 81) | ***P*** | |
| **Demographic** | Age (years) | 66.6 ± 9.9 | 67.9 ± 9.5 | ns | 65.9 ± 9.9 | | 64.3 ± 10.1 | ns | 66.7 ± 9.7 | | 67.6 ± 9.9 | ns | | 65.9 ± 9.9 | 64.2 ± 10.3 | ns | |
|  | Male sex (n,%) | 133 (60.7) | 51 (65.4) | ns | 158 (67.8) | | 51 (59.3) | ns | 133 (61.3) | | 51 (63.8) | ns | | 159 (66.8) | 50 (61.7) | ns | |
|  | BMI (kg/m²) | 27.2 ± 4.9 | 27.2 ± 4.5 | ns | 27.4 ± 4.8 | | 27.1 ± 4.3 | ns | 27.2 ± 4.5 | | 27.6 ± 4.4 | ns | | 27.2 ± 4.8 | 27.6 ± 4.4 | ns | |
| **Risk factors**  **(n,%)** | Hypertension | 144 (65.8) | 54 (69.2) | ns | 156 (67.0) | | 51 (59.3) | ns | 144 (66.4) | | 54 (67.5) | ns | | 160 (67.2) | 47 (58.0) | ns | |
|  | Diabetes | 39 (17.8) | 16 (20.5) | ns | 52 (22.3) | | 17 (19.8) | ns | 36 (16.6) | | 19 (23.8) | ns | | 56 (23.5) | 13 (16.0) | ns | |
|  | Smoking | 44 (20.1) | 18 (23.1) | ns | 51 (21.9) | | 16 (18.6) | ns | 46 (21.2) | | 16 (20.0) | ns | | 51 (21.4) | 16 (19.8) | ns | |
| **Lipoprotein(a)** | Lp(a) (nmol/L) | 19.5 [10.8-42.8] | 137 [108-219] | <0.001 | 21.1 [10.2-36.8] | | 141 [109-198] | <0.001 | 20.6 [12.2-52.8] | | 96 [44-188] | <0.001 | | 23.2 [11.5-53.5] | 114 [55-185] | <0.001 | |
|  | KIV domains (n) | 34.1 ± 8.3 | 25.5 ± 5.6 | <0.001 | 34.7 ± 7.9 | | 25.6 ± 5.6 | <0.001 | 35.5 ± 6.9 | | 22.1 ± 3.5 | <0.001 | | 35.7 ± 6.6 | 22.1 ± 3.2 | <0.001 | |
| **Lipids**  **(mg/dL)** | TC | 210 ± 30 | 215 ± 33 | ns | 215 ± 29 | | 215 ± 26 | ns | 210 ± 31 | | 213 ± 30 | ns | | 216 ± 28 | 214 ± 27 | ns | |
|  | LDL-C | 133 ± 24 | 136 ± 28 | ns | 138 ± 23 | | 140 ± 22 | ns | 134 ± 25 | | 134 ± 24 | ns | | 138 ± 23 | 138 ± 22 | ns | |
|  | HDL-C | 49 ± 11 | 49 ± 14 | ns | 49 ± 13 | | 49 ± 13 | ns | 49 ± 11 | | 48 ± 13 | ns | | 49 ± 14 | 48 ± 11 | ns | |
|  | TG | 141 ± 64 | 147 ± 69 | ns | 148 ± 65 | | 132 ± 55 | 0.064 | 135 ± 56 | | 161 ± 43 | 0.015 | | 146 ± 65 | 136 ± 56 | ns | |
| **Apolipopro-**  **teins (mg/dL)** | ApoA-I | 147 ± 24 | 147 ± 27 | ns | 146 ± 26 | | 146 ± 28 | ns | 147 ± 24 | | 147 ± 25 | ns | | 146 ± 28 | 147 ± 27 | ns | |
|  | ApoB100 | 134 ± 22 | 135 ± 23 | ns | 139 ± 23 | | 136.8 ± 22.2 | ns | 134 ± 22 | | 136 ± 23 | ns | | 139 ± 23 | 139 ± 20 | ns | |

*Values displayed as means ± SE or median [interquartile range] for continuous variables and as percentages for categorical variables. TC denotes total cholesterol, LDL-C low-density lipoprotein cholesterol, HDL-C high-density lipoprotein cholesterol, and TG Triglycerides. The BMI denotes body-mass index and is the weight in kilograms divided by the square of the height in meters.*

**Supplemental Table 5 - Distribution and Hazard Ratios of coronary and cerebrovascular events in the subgroup of 616 patients with carotid stenosis at baseline by treatment arm.**

|  | **Atorvastatin 80mg** | | | | | | **Placebo** | | | | | | | | |
| --- | --- | --- | --- | --- | --- | --- | --- | --- | --- | --- | --- | --- | --- | --- | --- |
| **By quartiles of Lp(a) concentrations** | | **< 84 nmol/L**  **(Q1-Q3)** | **≥84 nmol/L**  **(Q4)** | ***P*** | **HR** | **95%CI** | ***P*** | **< 84 nmol/L**  **(Q1-Q3)** | **≥84 nmol/L**  **(Q4)** | ***P*** | **HR** | **95%CI** | | ***P*** | |
|  |  | (n = 219) | (n = 78) |  |  |  |  | (n = 233) | (n = 86) |  |  |  |  |  |  |
| **Coronary events** | | 15 (6.8) | 10 (12.8) | *0.151* | 1.913 | (0.797-4.589) | *0.147* | 27 (11.6) | 8 (9.3) | *0.688* | 0.865 | | (0.376-1.943) | | *0.709* |
| **Cerebrovascular events**   - ***Ischemic Strokes*** | | 40 (18.3) | 19 (24.4) | *0.251* | 1.314 | (0.737-2.343) | *0.355* | 65 (27.9) | 16 (18.6) | *0.111* | 0.692 | | (0.385-1.242) | | *0.217* |
|  |  |  |  |  | 1.082 | (0.497-2.357) | *0.842* |  |  |  | 0.939 | | (0.489-1.806) | | *0.851* |
| **By quartiles of apo(a) size** | | **> 25.9 KIV**  **(Q2-Q4)**  (n = 217) | **≤ 25.9 KIV**  **(Q1)**  (n = 80) | ***P*** | **HR** | **95%CI** | ***P*** | **> 25.9 KIV**  **(Q2-Q4)**  (n = 238) | **≤ 25.9 KIV**  **(Q1)**  (n = 81) | ***P*** | **HR** | | **95%CI** | | ***P*** |
| **Coronary events** | | 13 (6.0) | 12 (15.0) | ***0.018*** | 2.974 | (1.275-5.935) | ***0.012*** | 27 (11.3) | 8 (9.9) | *0.838* | 1.128 | | (0.496-2.565) | | *0.774* |
| **Cerebrovascular events**   - ***Ischemic Strokes*** | | 40 (18.4) | 19 (23.8) | *0.327* | 1.278 | (0.719-2.271) | *0.403* | 65 (27.3) | 16 (19.7) | *0.187* | 0.940 | | (0.526-1.701) | | *0.853* |
|  |  |  |  |  | 1.227 | (0.575-2.616) | *0.597* |  |  |  | 1.064 | | (0.536-2.113) | | *0.859* |

**Supplemental Table 6 – IPTW adjusted Hazard Ratios of coronary, peripheral and cerebrovascular events by treatment arm.**

**Supplemental Table 7 – Fine-Gray adjusted Subdistribution Hazard Ratios of coronary, peripheral and cerebrovascular events by treatment arm, accounting for death as competitive risk.**

*Subdistribution Hazard Ratios (SHR) were adjusted for age, sex, type of entry event, time to entry event, BMI, non-HDL cholesterol levels, smoking, hypertension, and diabetes at baseline, accounting for death as a competitive risk.*

**Supplemental Figure 1 - Distribution of Lp(a) plasma concentrations in the study population (n=2,814).**


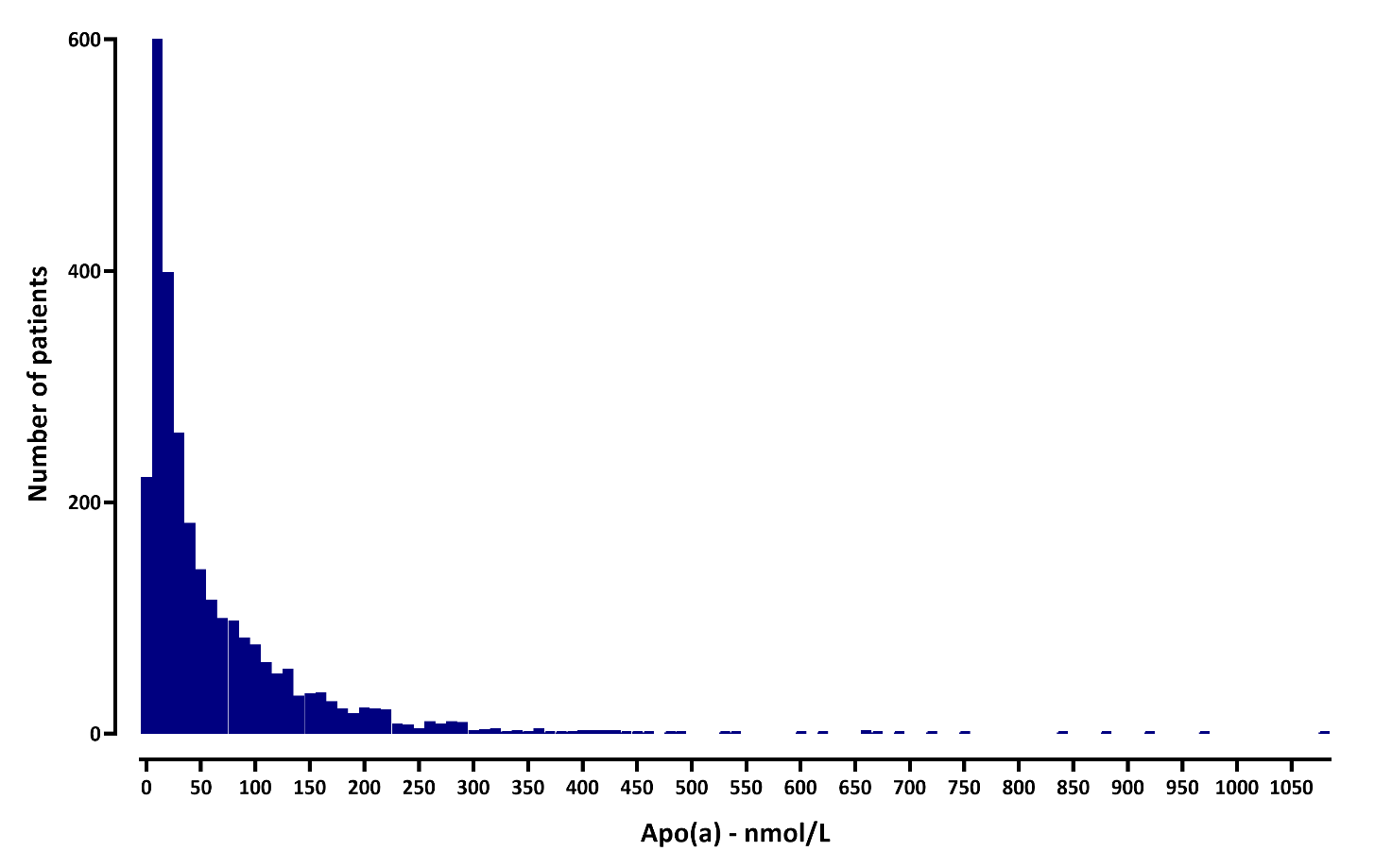


**Supplemental Figure 2 - Cumulative Incidence and Hazard Ratios of Peripheral Events by levels of Lipoprotein (a) plasma concentrations at baseline and by levels of apolipoprotein (a) size distribution during 6 years following randomization.** Kaplan-Meier estimates for peripheral events in patients randomized to placebo **(A)** or atorvastatin 80mg **(B)** with Lp(a) plasma concentrations below (Quartiles 1-3) or above (Quartile 4) 84 nmol/L at baseline. Kaplan-Meier estimates for peripheral events in patients randomized to placebo **(C)** or atorvastatin 80mg **(D)** with apolipoprotein (a) size below (Quartile 1) or above (Quartiles 2-4) 25.9 Kringle IV domains. Kaplan-Meier estimates and Hazard Ratios were adjusted for age, sex, BMI, type of entry event, time since entry event, , non-HDL cholesterol levels, smoking, hypertension, and diabetes at baseline. HR denotes hazard ratios, CI confidence intervals, and KIV Kringle IV domains.


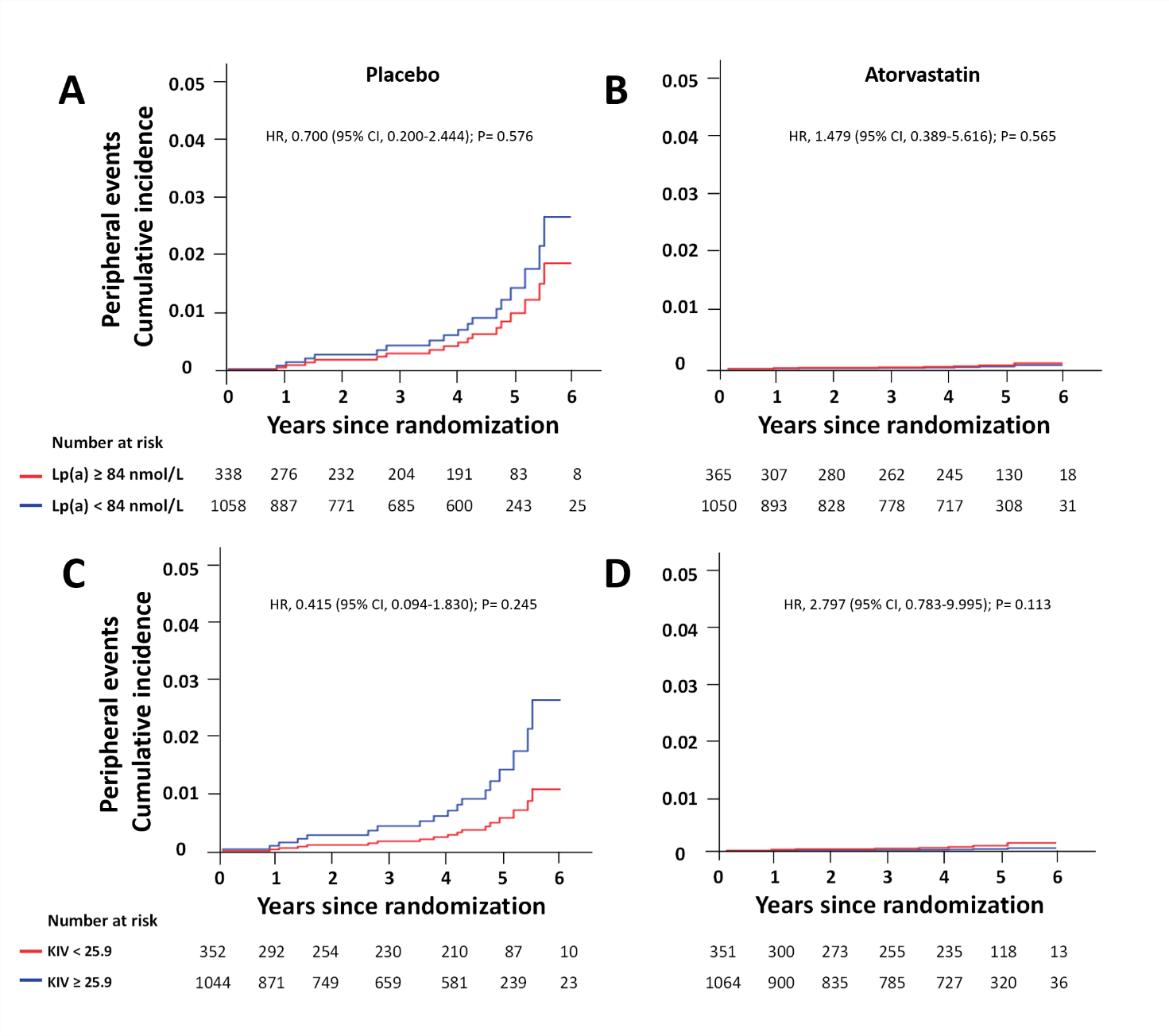


**References**

1. Blanchard V, Ramin-Mangata S, Billon-Crossouard S, et al. Kinetics of plasma apolipoprotein E isoforms by LC-MS/MS: a pilot study. J. Lipid Res. 2018;59:892–900.

2. Blanchard V, Chemello K, Hollstein T, et al. The size of apolipoprotein (a) is an independent determinant of the reduction in lipoprotein (a) induced by PCSK9 inhibitors. Cardiovascular Research 2022;118:2103-2111.

3. Blanchard V, Garçon D, Jaunet C, et al. A high-throughput mass spectrometry-based assay for large-scale profiling of circulating human apolipoproteins. J Lipid Res 2020;61:1128–1139.

4. Sillesen H, Amarenco P, Hennerici MG, et al. Atorvastatin Reduces the Risk of Cardiovascular Events in Patients With Carotid Atherosclerosis. *Stroke*. 2008;39(12):3297-3302
